# In Vitro Bone Marrow–Derived Dendritic Cells (BMDC) Generation for Antigen Presentation Assay

**DOI:** 10.21769/BioProtoc.5278

**Published:** 2025-04-20

**Authors:** Sudhakar Singh, Azeez Tehseen, Mohammed Shaaz Iqbal, Sharvan Sehrawat

**Affiliations:** Indian Institute of Science Education and Research, Mohali, India

**Keywords:** BMDCs, In vitro stimulations, OT1 cells, CFSE labeling, Co-culture, Cross antigen presentation

## Abstract

Dendritic cells (DC) are sentinel cells of the immune system that process and present antigens to activate T cells, thus serving to bridge the innate and adaptive immune systems. DCs are particularly efficient at cross-presentation whereby exogenously acquired antigens are processed and presented in context with MHCI molecules to activate CD8^+^ T cells. Assaying antigen presentation by DCs is a critical parameter in assessing immune functionality. However, the low abundance of bona fide DCs within the lymphoid compartments limits the utility of such assays. An alternative approach employing the culturing of bone marrow cells in the presence of factors needed for DC lineage commitment can result in the differentiation of bone marrow dendritic cells (BMDCs). This protocol details the process of in vitro generation of BMDCs and demonstrates their subsequent utility in antigen presentation assays. The protocol described can be adapted to various conditions and antigens.

Key features

• BMDCs can serve as surrogate antigen-presenting cells (APCs) for assessing in vitro and in vivo antigen presentation.

• Co-culture of antigen-stimulated BMDCs with CFSE-labeled T cells can help quantify the responsiveness of both the antigen presenters and responders.

• In vivo analysis of antigen presentation by BMDCs can be assessed using an adoptive transfer approach.

• CFSE labeling can help track in vivo the fate of adoptively transferred BMDCs as well as T cells.

## Graphical overview


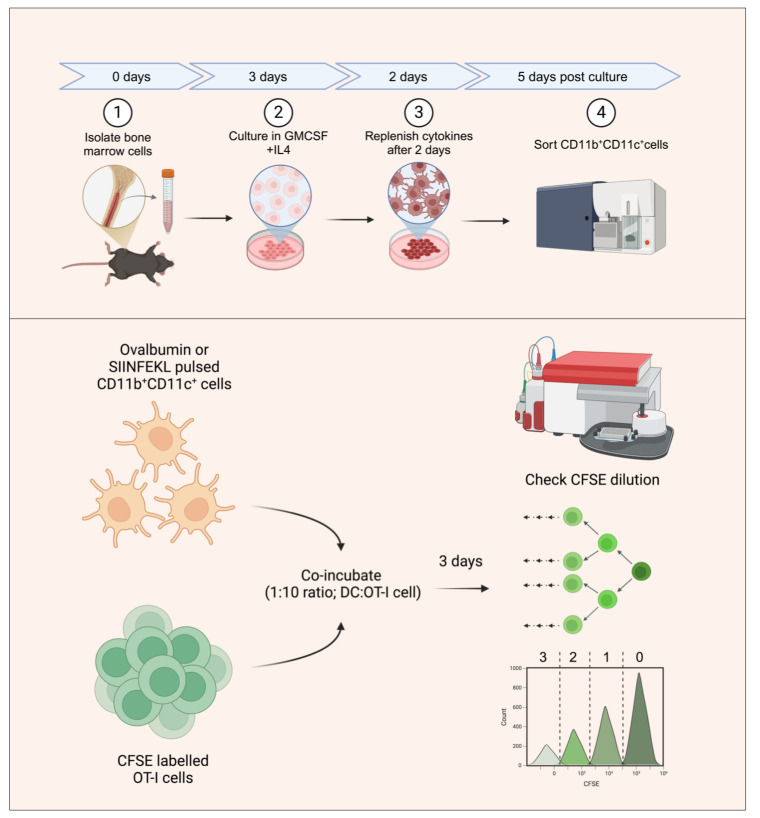


**Generating bone marrow dendritic cells (BMDCs) for in vitro antigen presentation assays.** The bone marrow precursors are cultured in the presence of interleukin 4 (IL-4) and granulocyte-macrophage colony-stimulating factor (GM-CSF) to generate DCs. BMDCs are then sorted as double-positive cells based on CD11b and CD11c staining. The sorted BMDCs are then pulsed with SIINFEKL peptide or ovalbumin protein and subsequently co-incubated with carboxyfluorescein succinimidyl ester (CFSE)-labeled OT-I T-cell receptor (TCR) transgenic cells with SIINFEKL-H-2K^b^ reactivity. The antigen presentation potential of the generated BMDCs is assayed by quantifying the frequencies of CFSE-diluting OT1 cells, with each peak representing a group of cells having undergone a different number of divisions.

## Background

An important breakthrough in immunology research was the development of in vitro functionality assays. While developing the mixed lymphocyte reaction assay, Mishell and Dutton made the unexpected finding that the lymphocytes by themselves were unable to produce an immune response and required glass-adhering accessory antigen-presenting cells (APCs) [1]. Subsequently, the work of Steinman and Cohn [2,3] established morphologically distinct accessory cell types, which they named dendritic cells (from the Greek *dendreon* meaning tree, owing to their tree-like projections). In 1978, Steinman and Nussenzweig developed the first antigen-presentation assay and showed that DCs present antigens to T cells to initiate immunity [4]. However, due to the extremely laborious procedure of purifying these cells, studies of DCs remained restricted to the laboratory of Steinman and his colleagues. Dendritic cell biology witnessed significant advances in the 1990s when Sallusto and Lanzavecchia developed an assay to differentiate DCs from blood monocytes by culturing them in the presence of granulocyte-macrophage colony-stimulating factor (GM-CSF) and interleukin (IL)-4 [5].

It is now well known that DCs are the main APCs involved in the recruitment of virus-specific CD8^+ ^T cells during an infection. Peptides derived from the processing of endogenous proteins or those acquired exogenously by the DCs are presented on the cell surface via the class I MHC molecules to specific CD8^+^ T cells [6,7]. The peptide-MHC-I complexes engage the specific TCR-expressing CD8^+ ^T cells (cytotoxic T lymphocytes, CTL), thus providing the first of three signals required for T-cell recruitment. The co-stimulatory receptor (B7.1/7.2) and ligand pair (CD28) are then engaged, which further enforces the T-cell activation program, which is fine-tuned by the generated cytokine milieu, also considered as signal II and III, respectively. The generation of an optimal CD8^+^ T-cell response is therefore contingent upon the proper functioning of multiple cellular processes within the DCs. These include 1) efficient uptake of exogenous antigens by the DCs, 2) processing of the internalized proteins into immunogenic peptides, 3) transport of the peptide-MHC complexes to the cell surface, 4) optimal expression of co-stimulatory molecules, and 5) optimal production of cytokines involved in T-cell priming. These events can efficiently be captured within the DCs by assaying their antigen presentation capability.

Here, we describe the generation of DCs from bone marrow precursors and subsequent analysis aimed at measuring their ability to prime antigen-specific CD8^+^ T cells by performing in vitro antigen presentation assays. To this end, we use the model CD8^+^ T-cell epitope, SIINFEKL (derived from the chicken egg ovalbumin, Ova_257-264_), as well as the complete ovalbumin protein to pulse bone marrow dendritic cells (BMDCs). The pulsed BMDCs were then co-incubated with OT1 cells (transgenic T cells having TCRs specific for the SIINFEKL epitope). The proliferation of the OT1 cells in response to stimulation with the pulsed BMDCs is then assayed using CFSE dilution. As such, the protocol described here provides a primer for studies aimed at assessing DC functionality in diverse conditions of health, disease, and aging and can be adapted to different epitopes and T-cell pairs.

## Materials and reagents

**Biological materials**

1. C57BL/6 mice (wild-type B6 mice, Jackson Laboratory, strain# 000664)

2. C57BL/6-Tg (TcraTcrb)1100Mjb/J (OT1 TCR transgenic mice, Jackson Laboratory, strain# 003831)

**Reagents**

1. RPMI (Gibco, catalog number: 31800-022)

2. Fetal bovine serum (FBS) (Gibco, catalog number: 26140-079)

3. Penicillin-streptomycin (Gibco, catalog number: 10378-016)

4. CFSE (Thermo Fisher Scientific, Invitrogen, catalog number: C34570)

5. Trypan blue 0.4% solution in Dulbecco’s phosphate buffered saline (HIMEDIA, catalog number: TCL046)

6. Ovalbumin protein (SERVA, catalog number: 11842.01)

7. SIINFEKL peptide (GL Biochem, catalog number: 181660)

8. Murine GM-CSF (Thermo Fisher Scientific, PeproTech, catalog number: 315-03-20UG, product format: 5 ng/mL in SFRPMI)

9. Murine IL4 (Thermo Fisher Scientific, PeproTech, catalog number: 214-14-20UG, product format: 5 ng/mL in SFRPMI)

10. Anti-Mouse CD11b (BioLegend, catalog number: 101205)

11. Anti-Mouse CD11c (BioLegend, catalog number: 117317)

12. Anti-Mouse CD8a (BioLegend, catalog number: 100733)

13. Mouse CD8 T-Cell Isolation kit (BioLegend, catalog number: 480008)

14. Sodium chloride (MERCK, EMPARTA, CAS: 7647-14-5)

15. Potassium chloride (HIMEDIA, CAS: 7447-40-7)

16. Potassium dihydrogen phosphate (SERVA, catalog number: 7778-77-0)

17. Disodium hydrogen phosphate (HIMEDIA, CAS: 7558-79-4)

18. Ammonium chloride (MERCK, CAS: 12125-02-9)

19. Sodium bicarbonate (SERVA, CAS: 144-55-8)

20. EDTA (MERCK, catalog number: 1.93312.1021)

21. Dimethyl sulfoxide (DMSO) (Thermo Fisher Scientific, catalog number: 85190)

**Solutions**

1. FACS buffer (see Recipes)

2. 1× phosphate-buffered saline (PBS) (see Recipes)

3. RBC lysis buffer (see Recipes)

4. RPMI (see Recipes)

5. CFSE stock solution (see Recipes)

6. CFSE working solution (see Recipes)

7. SIINFEKL peptide stock solution (see Recipes)

8. Ovalbumin stock solution (see Recipes)

**Recipes**

**1. FACS buffer**

**Table:** 

Reagent	Final concentration	Quantity or Final Volume
1× PBS	100 mL	100 mL
Fetal bovine serum	2%	2 mL

Prepare fresh each time before proceeding with the experiment.

**2. Phosphate buffered saline (PBS) (pH 7.4, for 1,000 mL)**

**Table:** 

Reagent	Final concentration	Quantity
Sodium chloride	137 mM	8 g
Potassium chloride	2.7 mM	0.201 g
Potassium dihydrogen phosphate	2 mM	0.260 g
Disodium hydrogen phosphate	10 mM	1.42 g

Store the PBS at 4 °C and warm it at room temperature before use.

**3. RBC lysis buffer (pH 7.3, for 750 mL)**

**Table:** 

Reagent	Final concentration	Quantity
Ammonium chloride	155 mM	6.218 g
Sodium bicarbonate	12 mM	0.756 g
EDTA	0.1 mM	27.918 mg

Store the RBC lysis buffer solution at 4 °C.

**4. RPMI (pH 7.3, for 500 mL)**

**Table:** 

Reagent	Final concentration	Quantity
Serum-free RPMI	1×	445 mL
Penicillin-streptomycin	1×	5 mL
FBS	1×	50 mL

Store the complete medium at 4 °C.

**5. CFSE stock solution (18 µL)**

**Table:** 

Reagent	Stock concentration	Quantity
CFSE dye	5 mM	NA
DMSO	1×	18 μL

Store the resuspended stock at -20 °C.

**6. CFSE working solution (500 μL)**

**Table:** 

Reagent	Final concentration	Quantity
CFSE stock solution	5 mM	0.5 μL
Serum-free RPMI	1×	500 μL

Prepare fresh each time before proceeding with the experiment.

*Note: The above working solution is shown for labeling ~10 million cells resuspended in 500 μL of serum-free RPMI.*

**7. SIINFEKL peptide stock solution (5 mL)**

**Table:** 

Reagent	Final concentration	Quantity
SIINFEKL peptide (lyophilized)	10 mg/mL	50 mg
DMSO	-	5 mL

Aliquot and store the peptide at -80 °C.

**8. Ovalbumin stock solution (5 mL)**

**Table:** 

Reagent	Final concentration	Quantity
Ovalbumin protein (lyophilized)	20 mg/mL	100 mg
RPMI	-	5 mL

Aliquot and store the stock solution at -80 °C.

**Laboratory supplies**

1. 100 mm Petri dish (Cole-Parmer, catalog number: 15971-43)

2. Round-bottom 96-well cell culture plate (Corning, catalog number: 3799)

3. 500 mL vacuum filter/storage bottle system 0.22 μm pore 33.2 cm^2^ PES membrane (Corning, catalog number: 431097)

4. 50 mL centrifuge tubes (Tarsons, catalog number: 546042)

5. 15 mL centrifuge tubes (Tarsons, catalog number: 546022)

6. 1.5 mL microcentrifuge tubes (Tarsons, catalog number: 500010)

7. 1,000 μL tips (Tarsons, catalog number: 521020)

8. 200 μL tips (Tarsons, catalog number: 521014)

9. 10 μL tips (Tarsons, catalog number: 521000)

10. Cell strainer, 70 μm (Cole-Parmer, catalog number: 04396-01)

11. Serological pipettes (Eppendorf, catalog number: 0030127730)

12. Cell scraper (Cole-Parmer, catalog number: 04396-53)

## Equipment

1. Biosafety cabinet (Labconco, model: Logic Class II, Type A2 Biosafety Cabinets)

2. CO_2_ incubator (Eppendorf, model: NB-170R)

3. Centrifuge (Eppendorf, model: AG 5810 R)

4. Inverted microscope (Olympus, model: IX53)

5. BD FACSAria^TM^ Fusion cell sorter (BD Biosciences)

6. BD Accuri C6 Flow cytometer (BD Biosciences)

7. Magnetic bars (Tarsons, catalog number: 4117)

8. Serological pipettes and micropipettes (Eppendorf Research Plus)

9. Cell counter (Marienfeld, hemocytometer 0640010)

10. Laboratory roller (Neuation, model: iRoll PR35)

## Software and datasets

1. BD FACSDiva software (Version 8.0.2)

2. GraphPad Prism (Version 9)

3. FlowJo (Version X)

4. BioRender (https://www.biorender.com/). The following figures were created using BioRender: Graphical overview, https://BioRender.com/uo9ctw9.

## Procedure

**A. Bone marrow isolation and processing of cells**

1. Euthanize C57BL/6 WT mice using a CO_2_ chamber following the procedure approved by the institutional animal ethics committee (IAEC).

*Note: Other IAEC-approved methods of euthanasia (e.g. cervical dislocation) may also be used.*

**Figure 1.**
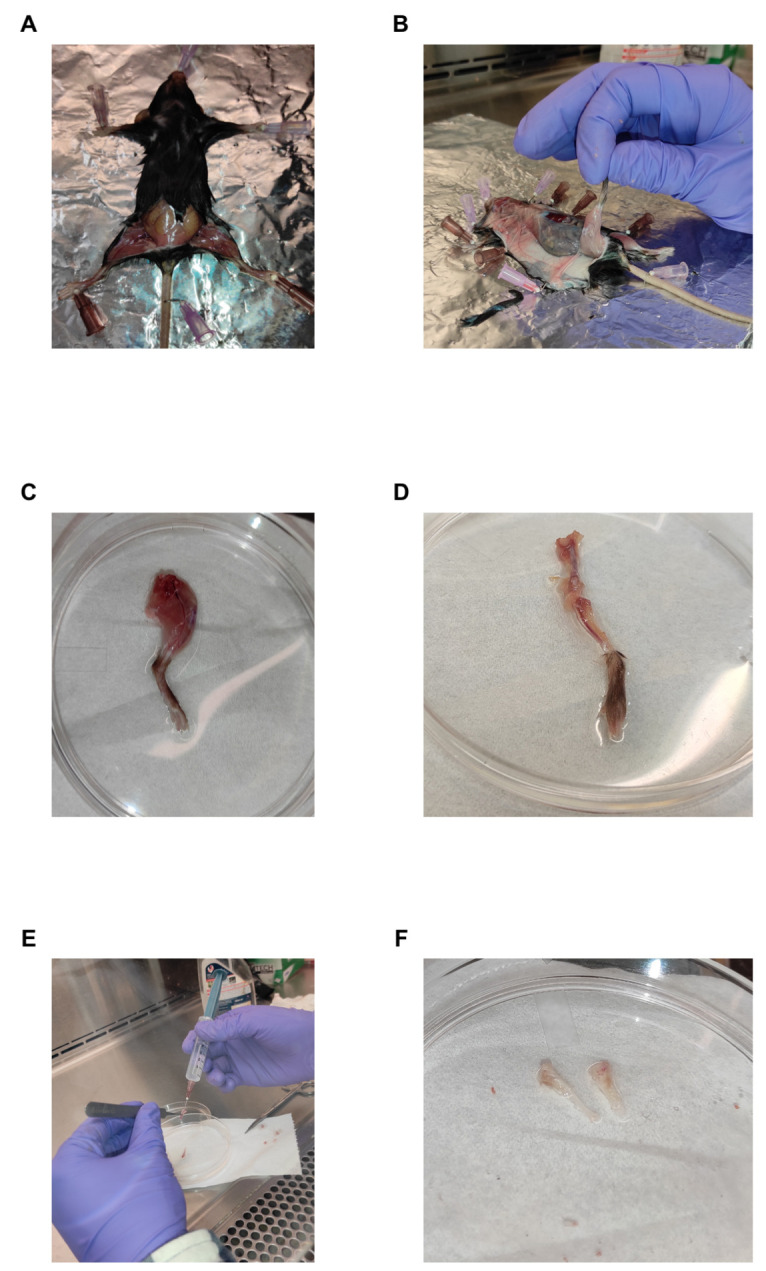
Representative images of each step involved in bone marrow isolation. (A) Mouse laid down in a supine position to cut the skin around the hip area. (B) Removal of skin around the hind legs. (C) Tibia and femur removed by cutting into the pelvis. (D) The femur and tibia are clearly visible upon the removal of muscles and fibrous tissues. (E) Flushing of the bones to obtain the bone marrow cells. (F) White bones clearly indicate proper flushing to remove the bone marrow. This can be compared with the red-colored bones in panel D.

2. Place the mouse in a supine position and cut open the skin in the hip area (Figure 1A).

3. Pull the skin toward the footpad of the hind legs (Figure 1B).

4. Remove the tibia and femur by cutting into the pelvis region (Figure 1C).

5. Remove the muscles and fibrous tissues from the bone by using scissors and forceps (Figure 1D).

6. Saturate a Kimwipe in 70% ethanol and wipe the bones to remove any remaining muscles and tissues.

*Note: The following steps are to be done under sterile conditions on ice.*

7. Add 10 mL of cold FACS buffer in a 100 mm cell culture dish.

8. Cut both ends at the epiphysis of the femur and tibia with a sharp scissor.

9. Hold the cut bones above the plate using forceps and flush out the bone marrows with cold FACS buffer in the culture plate using a 2 mL 25-gauge syringe until the bones appear white in color. This indicates complete flushing of the cellular contents from the bone marrow cavity (Figure 1E and F).

10. Transfer the cells into a 15 mL sterile Falcon and spin down at 300× *g* for 5 min at 4 °C.

11. Discard the supernatant and add 3 mL of ice-cold RBC lysis buffer, resuspend the cells gently by reverse pipetting, and incubate the Falcon tubes on ice for 3–4 min.

12. After 4 min, add 3 mL of RPMI in the Falcon tubes, mix gently, and spin down the cells at 300× *g* for 5 min at 4 °C.

13. Discard the supernatant and add 3 mL of RPMI to resuspend the pellet by gentle mixing using pipettes.

*Note: Make sure that pellets appear whitish. Traces of red color are indicative of incomplete RBC lysis. Repeat RBC lysis as mentioned above.*

14. Count the cells using a hemocytometer.

**B. In vitro BMDC generation**

1. To a 100 mm cell culture plate, add approximately 10–15 × 10^6^ bone marrow cells in 15 mL of RPMI supplemented with 5 ng/mL of GM-CSF and IL4.

*Note: The protocol can be adapted according to the requirements of the experiment. A single healthy mouse can yield 6–7 × 10^7^ bone marrow cells from femur and tibia bones. However, if the number of cells has to be changed, then it is suggested that the concentration of the cytokines also be optimized.*

2. Keep the plates in a humidified incubator at 5% CO_2_ at 37 °C for 2 days.

3. After 2 days, replace 5 mL of fresh RPMI supplemented with 5 ng/mL of GM-CSF and IL4 with the equivalent volumes of the existing culture medium in each plate.

*Note: Replacing fresh medium with a fraction of the existing medium ensures minimal loss of the accumulated factors within the cultures of differentiating cells.*

**Figure 2.**
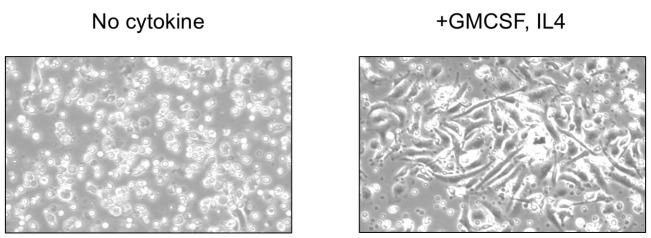
Bone marrow cells upon culture in medium devoid of (left panel) or supplemented with granulocyte-macrophage colony-stimulating factor (GM-CSF) + interleukin (IL) 4 (right panel) to differentiate into bone marrow dendritic cells (BMDCs). The differentiation of BMDCs is confirmed by the presence of cells with dendritic projections within the medium supplemented with GM-CSF and IL4.

4. On the sixth day of initial seeding, cells with dendritic projections should be observed in the culture dishes (Figure 2).

5. Discard all suspended cells along with the supernatant.

*Note: The dendritic cells tend to adhere to the Petri dish, whereas the cells in suspension are undifferentiated or non-viable cells. The unadhered cells are therefore discarded to enrich the population of dendritic cells.*

6. Add 5 mL of RPMI to the plates and gently scrap the adhered cells using a cell scraper.

*Note: Confirm the detachment of cells by viewing them under a microscope.*

7. Collect the cells in 15 mL Falcon tubes and spin down the cells at 300× *g* for 5 min at room temperature.

8. Resuspend the cells in 3 mL of RPMI. The cells are now ready to be stained for sorting.

**C. Sorting CD11c^+^ and CD11b^+^ BMDCs**

1. Aliquot 500 μL of the resuspended cells in three equal parts in separate microcentrifuge tubes.

2. Label one tube as unstained, one tube as CD11b-only, and one tube as CD11c-only.

3. Add the required volume of the anti-mouse CD11b or CD11c antibody to the CD11b or CD11c-only tubes, respectively.

*Note: The cells stained only with CD11b and CD11c antibodies will be used for setting up the instrument and as fluorescent-minus-one (FMO) gating controls.*

4. Stain the remaining cells in the 15 mL Falcon tube by adding the required volume of mouse anti-CD11b-FITC (1:400 dilution) and anti-CD11c-PE-Cy7 (1:300 dilution) antibodies. This is your master sample for sorting CD11c^+^CD11b^+^ BMDCs.

5. Leave the stained cells in the dark on ice for 45 min.

6. Add to the Falcon tube 10 mL of FACS buffer and wash the cells by spinning down at 300× *g* for 5 min at 4 °C.

7. After discarding the supernatants, resuspend the cells in 1 mL of RPMI and strain the cells using a 70 μm cell strainer to remove any clumps.

*Note: The passing of the cells through a cell strainer is a very important step to avoid clogging of the fluidics in the sorter.*

8. Run the staining controls (unstained sample and FMOs) and adjust the voltage so that the populations of interest are properly visible.

*Note: We have used BD FACSAria Fusion for the sorting of the CD11b^+^CD11c^+^ cells. A similar approach of first running the staining controls and FMOs to adjust the voltages and gates has to be applied irrespective of the type of instrument being used. Discuss the experimental setup with the core facility or instrument operator to know what is required to perform the experiment on a particular instrument.*

**Figure 3.**
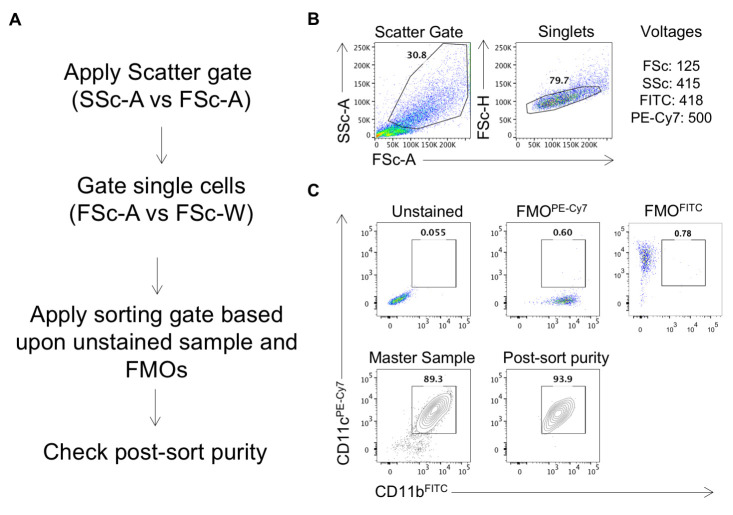
Sorting strategy for the sorting of CD11b^+^CD11c^+^ dendritic cells. (A) Following the gating of live cells and singlets based upon scattering (B), apply the sorting gates for your markers of interest using the fluorescent-minus-one (FMO) controls. Sort the cells and, upon completion, perform a post-sort purity check by acquiring a small number of the sorted cells (C).

9. Apply the sorting gate according to the FMOs.

10. Sort the double positive (CD11c^+^CD11b^+^) cells. See Figure 3 for the sorting strategy and representative FACS plots.

11. Run a post-sort purity check to confirm the purity of sorted cells.

**D. OT1 cell isolation**

**Figure 4.**
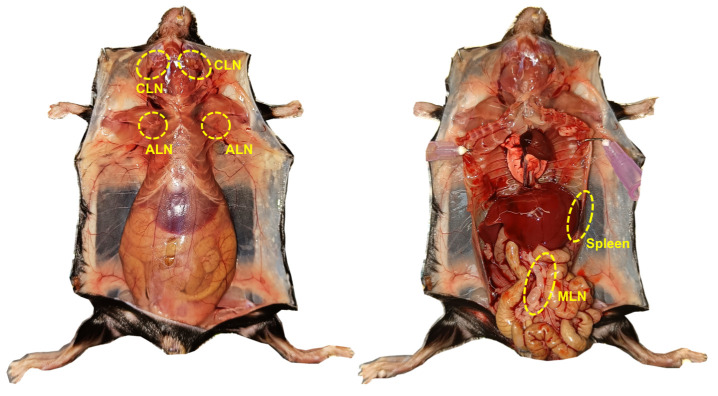
Anatomical locations of the lymph nodes isolated for the purification of OT1 cells. CLN: Cervical lymph node; ALN: axillary lymph node; MLN: mesenteric lymph node and spleen.

1. Euthanize OT1 TCR transgenic mice using a CO_2_ chamber following the procedure approved by the institutional animal ethics committee (IAEC).

2. Isolate cervical, axillary, and mesenteric lymph nodes (LNs) and spleen (Figure 4).

3. Gently crush the LNs and spleens using the soft end of the plunger of a 3 mL syringe in a 70 μm cell strainer kept in a 100 mm Petri dish containing 2 mL of cold RPMI. Add ~8 mL of RPMI and transfer the cells to a 15 mL Falcon tube.

4. Pellet the cells by spinning down at 300× *g* at 4 °C for 5 min.

5. Discard the supernatant and perform RBC lysis as mentioned in section A.

6. Spin down the cells at 300× *g* for 5 min at 4 °C and discard the supernatant.

7. Resuspend the cells in 10 mL of RPMI. Wash the cells by spinning down at 300× *g*, similar to the previous step.

8. Resuspend the cells in 500 μL of RPMI.

9. Count the cells and purify CD8^+^ T cells by MACS procedure following the manufacturer’s instructions:

a. Add the recommended volume of the biotin-conjugated antibody cocktail to the resuspended cells and incubate as per the manufacturer’s instructions.

b. Wash the cells and resuspend them in RPMI to remove any unbound antibodies as they might interfere with the magnetic bead binding.

c. Add the recommended volume of the streptavidin-conjugated magnetic beads to the resuspended cells and incubate as per the manufacturer’s instructions.

d. Isolate the CD8^+^ T cells (this step may vary according to the kit being used).

*Note: We have provided an overview of the protocol of MACS purification; however, we highly recommend the reader to confer with the manufacturer’s protocol in order to have optimum yield.*

10. Wash the MACS-purified cells once by spinning at 300× *g* for 5 min.

11. Finally, resuspend the cells in 2 mL of serum-free media and count the cells.

*Note: A single homozygous OT1 mouse may yield 1–1.5 × 10^7^ cells*.

12. The cells are now ready for CFSE labeling.

**E. CFSE labeling of OT1 T cells**

*Note: These steps must be performed in the dark under sterile conditions.*

1. Take approximately 10 million cells in 500 μL of serum-free RPMI in 15 mL Falcon tubes.

2. Dilute CFSE to the working concentration (5 μM) by adding 0.5 μL of the stock solution (5 mM) to 500 μL of serum-free RPMI in a separate 1.5 mL microcentrifuge tube. Mix by vortexing.

3. Add the working solution of CSFE dropwise to the cells with gentle mixing of cells.

4. Incubate the Falcon at room temperature for 10 min.

5. Following incubation, add an equal volume of serum to quench the free dye.

6. Wash the cells by spinning at 300× *g* for 5 min.

7. Resuspend the cells in 1 mL of RPMI.

8. Keep the cells in the dark on ice.

9. The cells are now ready for setting up antigen presentation.

**F. In vitro stimulation of BMDCS and co-culture with OT1 cells**

1. Add varying concentrations of either the SIINFEKL peptide or whole ovalbumin protein to 2 × 10^4^ of BMDCs for 45 min and 2 h, respectively, in a 96-well U-bottom plate:

a. Dilute the SIINFEKL stock solution in RPMI to a working stock concentration of 1 mg/mL and add the required volume of this working stock to the DCs for pulsing.

b. Dilute the ovalbumin stock solution to a concentration of 200 μg/mL in RPMI and add the required volume to the DCs for pulsing.

*Note: The main goal of the assay is to check the capability of the DCs to prime CD8^+^ T cells; to this end, any logical range of concentrations can be used. We have used 1–100 μg for the SIINFEKL peptide and 1–5,000 ng for the ovalbumin protein. Readers are suggested to optimize their own range of concentrations as per their requirements and experimental setup.*

2. After incubation, wash the antigen-pulsed DCs by centrifuging the plates at 300× *g* for 5 min at room temperature.

*Note: The plate is flicked to discard the old medium before resuspending the cells.*

3. Resuspend the cells in 100 μL of RPMI and wash the cells as above.

4. Resuspend the wells using 100 μL of RPMI and add 2 × 10^5^ OT1 cells per well.

5. Analyze the stained CD8^+^ T cells for CFSE dilution after 72 h of incubation.

**G. Staining of cells and sample acquisition using a flow cytometer**

1. Stain the cells in the dark at 4 °C by adding anti-mouse CD8α antibody (1:100 dilution) for 30 min.

2. Wash the cells twice by spinning at 300× *g* for 5 min.

3. Resuspend the cells in 200 μL of FACS buffer and acquire the samples using a flow cytometer.

*Note: We have used a BD Accuri C6 flow cytometer for data acquisition. This cytometer does not require the setting of voltages; however, we did place a cutoff of 500,000 on the FSc-H parameter in order to minimize debris.*

4. Analyze the division of CFSE-labeled cells.

## Data analysis

The analysis of the data requires some experience with flow cytometry analysis. We have performed the analysis using FlowJo software. For a detailed account of the analysis and gating strategies, readers can refer to the main and supplementary figures in reference [8].

**A. Gating strategy**

**Scatter gate (FSC vs. SSC)**

**Figure 5.**
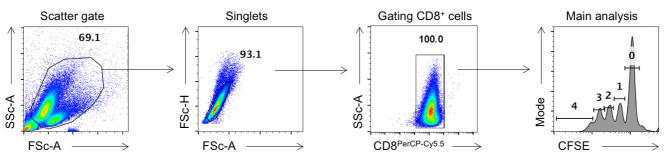
Representative gating strategy for analysis of antigen presentation assays. Cells were first gated based upon forward and side scatter to exclude debris, followed by singlet gating to eliminate doublets. The OT1 cells were then identified by gating the CD8^+^populations. Proliferation of the OT1 cells was assessed by analyzing CFSE dilution, with individual peaks representing successive rounds of cell division.

The scatter gate identifies the population of interest based on cell size (FSc) and granularity (SSc). This step excludes debris and focuses on intact cells. Gate the cells to exclude debris as shown in Figure 5.

**Singlets gate (FSC-A vs. FSC-H)**

This step ensures that only single cells are included in the analysis, as doublets can skew results:

• The FSc-A (area) and FSc-H (height) measure light scatter and the peak light intensity, respectively.

• Single cells typically have a linear relationship between FSc-A and FSc-H and therefore appear diagonally in the cluster.

• Doublets and aggregates have higher FSc-A values relative to the FSc-H and deviate from the diagonal.

• Draw a narrow diagonal gate to include only singlets as shown in Figure 5.

• Exclude any events outside this diagonally drawn gate (e.g., higher FSc-A values for the same FSc-H).

**Gating CD8^+^ T cells**

Since the division of CD8^+^ T cells is to be assayed, gating on CD8^+^ T cells before analyzing CFSE dilution is required. However, as is evident from Figure 5, at this time point, all cells are CD8^+^ due to their extensive proliferation.

**Gating CFSE dilution peaks**

We can then quantify cell proliferation by gating individual peaks of CFSE dilution. Each peak corresponds to the generation of dividing cells, with successive divisions showing reduced CFSE content.

• The **parental peak** (non-divided cells) will have the highest fluorescence intensity (as shown in Figure 5).

• Successive peaks to the left represent cells that have divided once, twice, three times, etc. (Main analysis, Figure 5).

• Gate each peak, starting with the brightest peak (rightmost peak, non-divided cells) and moving to the left for subsequent generations. Copy exactly the same gating and paste in the files for all the samples for a comparative analysis.

*Note: The number of visible peaks will depend on the number of cellular divisions.*

## Validation of protocol

We now present a sample antigen presentation assay for the validation of the protocol. To this end, the generated BMDCs were pulsed with graded concentrations of either SIINFEKL peptide or the complete ovalbumin protein. The cells were then co-incubated with CFSE-labeled OT-I cells in a 1:10 ratio (BMDC: OT-I). The division of the OT-I cells was then assessed by checking CFSE dilution. Three technical replicates were taken for each concentration of the peptide or the Ova-protein. A total of 50,000 cells were acquired per sample. Data are shown in Figure 5. A representative FACS plot is shown for the indicated concentration of the peptide or the Ova-protein.

**Figure 6.**
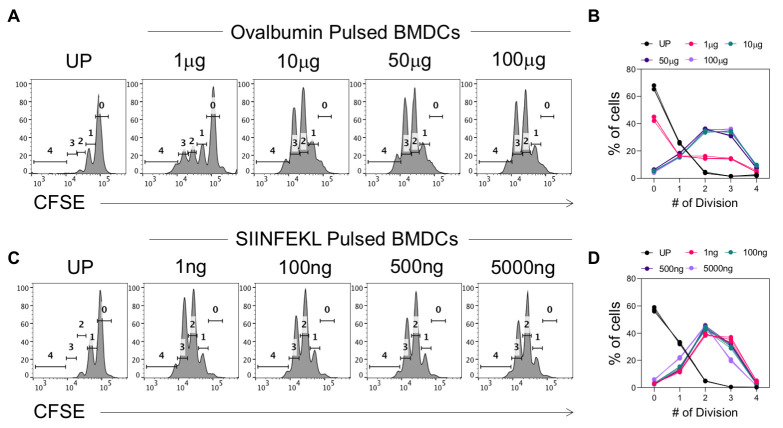
In vitro antigen presentation assay. Bone marrow dendritic cells (BMDCs) were generated and pulsed with the indicated doses of SIINFEKL peptide or ovalbumin protein for 45 min and 2 h, respectively. BMDCs were then sorted and co-incubated with CFSE-labeled OT-I cells for 72 h, following which CFSE dilution by the OT-I cells was checked. (A) Representative histogram plots for analysis of CFSE dilution by OT-I cells co-incubated with BMDCs pulsed with the indicated doses of ovalbumin protein. Un-pulsed BMDCs (UP) were taken as a control to gate the undivided cells. (B) Line plot summarizing the frequency of cells having undergone the indicated number of divisions. (C) Representative histogram plots for analysis of CFSE dilution by OT-I cells co-incubated with BMDCs pulsed with the indicated doses of SIINFEKL peptide. (D) Line plot summarizing the frequency of cells having undergone the indicated number of divisions. Each line and datapoint represent an individual technical replicate (n = 3), with each color representing a different concentration.

While a low dose of ovalbumin stimulation (1 μg, Figure 6A and B) shows lower frequencies of cells having undergone four divisions, the frequency of cells having undergone multiple divisions is uniform in the case of co-incubation with SIINFEKL-pulsed DCs (Figure 6C and D). Accordingly, while approximately 40% of the cells are still at the first peak of division in the case of a low dose of ovalbumin, this is not the case for a low dose of SIINFEKL peptide. This is because while the complete ovalbumin protein has to be processed and presented, the SIINFEKL epitope does not undergo the same. Therefore, trace amounts of the peptide can strongly induce an antigen-specific response, whereas low doses of the complete protein cannot do so, as is evident from Figure 6A and B.

This protocol or parts of it has been used and validated in the following research article(s):

• Singh et al. [8]. Rab8a restores diverse innate functions in CD11c^+^CD11b^+^ dendritic cells from aged mice. *Nature Communications* (Figure 2b and c, Figure 5f and g, Figure 6b and c, and Figure 7f–k.)

• Sarkar et al. [9]. Myeloid derived suppressor cells potentiate virus-specific memory CD8^+^ T cell response. *Microbes and Infection* (Supplementary Figure S1, Panel B).

## General notes and troubleshooting

**General notes**

1. This protocol explains the methodology to perform the expansion of CFSE-labeled OT1 cells using antigen-pulsed BMDCs.

2. The use of GM-CSF and IL-4 for the generation of BMDCs has been described in this protocol. The type and quality of the DCs vary according to the concentration of cytokines and the duration of incubation. Accordingly, variability in the differentiating populations between experiments is expected. Therefore, we strongly suggest that the sorting of the generated BMDCs should be done before setting up co-culture assays to ensure that only CD11b^+^CD11c^+^ cells are present within the population.

3. Even though we have stained the BMDCs with mouse anti-CD11c and anti-CD11b for sorting of the cells, the inclusion of other markers is preferable to avoid the contamination of neutrophils (Gr1, Ly6G), macrophages (Ly6C, F4/80), and NK cells (NK1.1 for B6 mice) from the BMDCs. Additionally, other markers could be included to specifically sort different lineages of the DCs such as cDC1 (CD8a^+^CD11c^+^) or cDC2 (CD8a-CD11b^+^CD11c^+^).

4. This protocol explains the pulsing of BMDCs for 45 min and 2 h with SIINFEKL peptide or the complete ovalbumin protein, respectively. When performing the cross-antigen presentation with different complex antigenic proteins, one should optimize the concentration of the stimulant and the timing of stimulations to obtain interpretable results.

5. Along with un-pulsed controls wherein no antigen-pulsed APCs are co-cultured with the CFSE-labeled T cells, it is suggested to keep some wells with only the CFSE-labeled OT1 cells, which serve as a control for non-proliferating cells.
